# ‘Resistance Mixtures’ Reduce Insect Herbivory in Strawberry (*Fragaria vesca*) Plantations

**DOI:** 10.3389/fpls.2021.722795

**Published:** 2021-09-23

**Authors:** Tuuli-Marjaana Koski, Sanne de Jong, Anne Muola, Daniel B. Amby, Erik Andreasson, Johan A. Stenberg

**Affiliations:** ^1^ Department of Plant Protection Biology, Swedish University of Agricultural Sciences, Alnarp, Sweden; ^2^ Section of Ecology, Department of Biology, University of Turku, Turku, Finland; ^3^ Department of Plant and Environmental Sciences, University of Copenhagen, Copenhagen, Denmark

**Keywords:** genotypic diversity, plant resistance, cultivar mixture, associational resistance, IPM

## Abstract

The transition toward more sustainable plant protection with reduced pesticide use is difficult, because there is no “silver bullet” available among nonchemical tools. Integrating several plant protection approaches may thus be needed for efficient pest management. Recently, increasing the genetic diversity of plantations *via* cultivar mixing has been proposed as a possible method to reduce pest damage. However, previous studies have not addressed either the relative efficiency of exploiting cultivar mixing and intrinsic plant herbivore resistance or the potential utility of combining these approaches to increase cropping security. Here, using a full factorial experiment with 60 woodland strawberry plots, we tested for the relative and combined effect of cultivar mixing and intrinsic plant resistance on herbivore damage and yield. The experiment comprised two levels of diversity (“high” with 10 varieties and “low” with two varieties) and three levels of resistance (“resistant” comprising only varieties intrinsically resistant against strawberry leaf beetle *Galerucella tenella*; “susceptible” with susceptible varieties only; and “resistance mixtures” with 50:50 mixtures of resistant and susceptible varieties). The experiment was carried out over two growing seasons. Use of resistant varieties either alone or intermixed with susceptible varieties in “resistance mixtures” reduced insect herbivory. Interestingly, resistant varieties not only reduced the mean damage in “resistance mixtures” by themselves being less damaged, but also protected intermixed susceptible varieties *via* associational resistance. The effect of higher genetic diversity was less evident, reducing herbivory only at the highest level of herbivore damage. In general, herbivory was lowest in plots with high diversity that included at least some resistant varieties and highest in low diversity plots consisting only of susceptible varieties. Despite this, no significant difference in yield (fruit biomass) was found, indicating that strawberry may be relatively tolerant. Our results demonstrate that combined use of high genetic diversity and resistant varieties can help reduce pest damage and provide a useful tool for sustainable food production. “Resistance mixtures” may be particularly useful for sensitive food crops where susceptible varieties are high yielding that could not be completely replaced by resistant ones.

## Introduction

Modern crop production is heavily dependent on synthetic pesticides, which are known to interfere with important ecosystem services such as pollination and biological control (Ma et al., 2000; Stanley et al., 2015) and may impair human health (McCauley et al., 2006). As extensive use of pesticides may also induce evolution of pesticide resistance, thereby reducing their efficiency, there is an urgent need to increase the use of alternative plant protection methods to secure global food production. Integrated pest management (IPM, see Table 1) provides a holistic plant protection approach by combining multiple plant protection methods (Peterson et al., 2016; Stenberg, 2017). The use of pest-resistant cultivars may be the most extensively studied and applied method (Tooker and Frank, 2012; Peterson et al., 2016), but while improving intrinsic plant resistance can partly reduce pest damage (and thus pesticide use), combinations with other methods are required for sufficient protection.

**Table 1:** Explanation of key terms used in this article.

Term	Explanation
Integrated pest management (IPM)	IPM. Use of multiple pest control methods to reduce the use of chemical pesticides
Intercropping	Cultivation of two or more crop species together
Cultivar mixture	Cultivation of several cultivars or varieties of the same crop species in the same plantation. In this experiment: high diversity treatment consisted of 10 varieties
Plantation	Field where commercial corps are grown
Plot	In this experiment, an experimental unit growing 40 plants, consisting either two or 10 genotypes that are either homogenously resistant, susceptible or their mixture
Plot resistance	In this experiment, cultivation of resistant or susceptible varieties alone or in a 50:50 mixture, resulting in three levels of plot resistance: resistant only, susceptible only or resistance mixture
Plant resistance	In this experiment, intrinsic resistance of a plant or variety to chewing herbivores
Associational resistance	Resistance or other characteristics of a plant can help to reduce pest damage in neighboring plants. For example, the presence of intrinsically resistant varieties may reduce the damage to susceptible varieties growing in the same plot
Associational susceptibility	Characteristics of another species or variety can increase pest damage in the neighboring plants. For example, the presence of susceptible plants may attract more herbivores, increasing the damage in mixed plots
Resistance mixture	Simultaneous cultivation of resistant and susceptible varieties. In this study, plots where resistant and susceptible varieties are grown together in a 50:50 mixture

In recent decades, it has become clear that increased crop diversity in agricultural systems can mitigate damage by pests and pathogens, improve yield size and stability, and enhance ecosystem services (Altieri, 1999; Zhu et al., 2000; Lin, 2011; Mulumba et al., 2012; Moreira et al., 2012; Isbell et al., 2017; Dainese et al., 2019; Yang et al., 2019) and it has become a standard recommendation in IPM. Increasing the diversity of agricultural systems can be accomplished by either increasing interspecies diversity *via* intercropping several crop species or by increasing intraspecies diversity *via* mixing several cultivars. There are several examples showing how increased interspecific diversity can reduce pest damage (Jactel and Brockerhoff, 2007; Letourneau et al., 2011; Tooker and Frank, 2012; Field et al., 2020, but see Vehviläinen et al., 2006). Although intercropping has proven to be a useful pest management tool, it has not been widely applied due to economic and practical reasons (Lin, 2011; Letourneau et al., 2011; Tooker and Frank, 2012). Cultivar mixing (Table 1), on the other hand, requires fewer changes in agricultural practices compared to intercropping, and it is, therefore, probably a more practical and financially feasible approach to improve the genetic diversity of plantations (Lin, 2011).

Similar to intercropping, cultivar mixing should decrease herbivore damage *via* multiple complementary mechanisms: According to the “variance in edibility,” “resource concentration,” and “dilution effect” hypotheses, the herbivore performance and population size of herbivores should be reduced in cultivar mixtures. This is because the increased heterogeneity of host plant populations should disrupt successful host location, increase the likelihood of leaving the food patch (Root, 1973; Thiéry and Visser, 1987; Peacock and Herrick, 2000; Duffy, 2002; Civitello et al., 2015; Field et al., 2020), and/or force herbivores to consume nonpreferred plant cultivars as well (diet mixing; Peacock and Herrick, 2000; McArt and Thaler, 2013), thereby reducing the likelihood of the herbivores reaching outbreak levels. However, to date nearly all studies about cultivar mixing focus on its effects on pathogens (Zhu et al., 2000; Mulumba et al., 2012; Yang et al., 2019; but see Peacock and Herrick, 2000; Ninkovic et al., 2002; and Doherty et al., 2019 for studies of herbivores). For example, higher intraspecies variation obtained by cultivar mixing has been shown to protect banana (*Musa* spp.) and common bean (*Phaseolus vulgaris* L.) plantations against pathogens, especially in areas with high disease risks (Mulumba et al., 2012). Meanwhile, the effects of intraspecific plant variation against damage caused by leaf chewing herbivores have received less attention.

Besides the mitigating effect increased diversity of plantation has on pests and pathogens, responses of pests to plantation’s diversity may depend on the characteristics of the cultivars involved – that is, their functional traits, rather than the genetic diversity *per se* (Ninkovic et al., 2002; Barton et al., 2015; Dahlin et al., 2018). For example, Ninkovic et al. (2002) showed that certain combinations of barley (*Hordeum vulgare* L.) cultivars reduced aphid acceptance more than others, although the underlying plant traits were not identified. The importance of having resistant neighbors has previously been explored within the frameworks of associational resistance and susceptibility (Tahvanainen and Root, 1972; White and Whitham, 2000; Ninkovic et al., 2002; Barbosa et al., 2009; Letourneau et al., 2011; Table 1). Most examples of associational resistance against herbivores have dealt with interspecific plant neighbors, but theoretically similar mechanisms could protect susceptible plant genotypes growing in the vicinity of resistant genotypes of the same plant species. Such effects have been demonstrated for phytopathogens; for example, susceptible rice cultivars intermixed with resistant cultivars experienced reduced damage by fungal pathogens, leading to increased yields of the susceptible cultivars (Zhu et al., 2000). Cultivating resistant and susceptible cultivars within the same field (here termed “resistance mixing,” Table 1) should, in theory, be a particularly beneficial strategy against insect herbivores as this approach simultaneously utilizes two complementary mechanisms for reducing herbivore attraction and performance. First, increasing the number of cultivars will increase genotypic diversity and the complexity of the habitat. Second, high intrinsic resistance of some cultivars not only interferes with herbivore performance (War et al., 2012) but also increases the functional diversity of the plantation when intermixed with susceptible cultivars (Bustos-Segura et al., 2017).

These and similar results suggest that the efficiency of cultivar mixing in reducing herbivory could be amplified if the traits responsible for resistance to herbivores are identified and optimized. However, the relative importance of plant genetic diversity and intrinsic plant resistance for reducing herbivory is not well known. Here, in a large-scale field experiment, we tested whether genetic diversity alone (cultivar mixing) and in combination with intrinsically resistant plants (i.e., “resistance mixture”) affects damage inflicted by free-living herbivores and yield in woodland strawberry plots by using 10 genotyped varieties (hereafter, referred as variety) preciously screened for their herbivore resistance (Stenberg, 2014; Muola et al., 2017; Weber et al., 2020a). More specifically, we manipulated both the functional diversity (i.e., the level of intrinsic resistance of individual plants and plots) and the genetic diversity (i.e., the number of plant genotypes per plot), which have rarely or never been systematically manipulated simultaneously. We hypothesized that increasing genetic and functional diversity should reduce herbivore damage, both alone and synergistically. Furthermore, we expected that associational resistance in resistance mixture plots would reduce herbivory on susceptible plants, that is, that the presence of resistant varieties would reduce damage on and increase yield of their susceptible neighbors compared to when susceptible varieties are grown alone (homogenously susceptible), and that this effect would increase with the number of varieties used.

## Materials and Methods

### Plant Material and Handling

We used woodland strawberry (*Fragaria vesca* L., Rosaceae) as a model species to study the relative importance of plant genetic diversity and intrinsic plant resistance on herbivore damage. Woodland strawberry is cultivated as a horticultural crop in parts of Europe (e.g., Scandinavia and Sicily), but is also widely used as a model species in plant science. In plant breeding, it plays an important role as a crop wild relative contributing wild genetic resources to new cultivars of garden strawberry (*Fragaria×ananassa*; Hancock and Luby, 1993; Egan et al., 2018). Woodland strawberry has considerable intraspecific variation in traits conferring resistance (antibiosis and antixenosis) to the strawberry leaf beetle (*Galerucella tenella* L.; Stenberg, 2014; Muola et al., 2017; Weber et al., 2020a). Despite that the main host of this oligophagous beetle is the meadowsweet (*Filipendula ulmaria* L., Maxim.), it is a common pest of garden strawberry (*F×ananassa*) in Northern Europe (Stenberg and Axelsson, 2008). We previously screened 86 woodland strawberry genotyped varieties for antibiosis and antixenosis against *G. tenella* (Weber et al., 2020a) and identified several primary and secondary metabolites that are associated with plant resistance (Weber et al., 2020b). Although the exact resistance mechanism requires further experiments, these results indicate the presence of a true intrinsic resistance. Therefore, we use these varieties as a general model to represent variation in plant resistance against leaf chewing herbivores. For this study, we chose the 10 most resistant and 10 most susceptible varieties, identified in Weber et al. (2020a).

**Figure 1:**
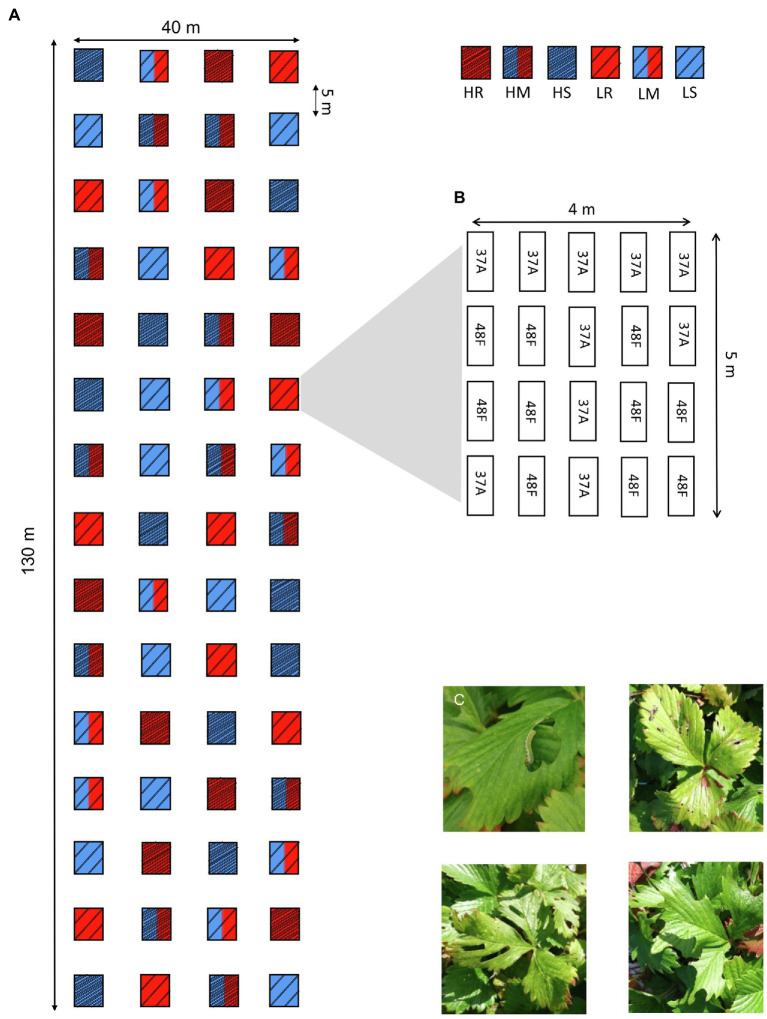
**(A)** Schematic representation of the experimental design testing the effects of plot diversity (i.e., cultivar mixture) and resistance on chewing herbivore damage in woodland strawberry (*Fragaria vesca*). H= high diversity i.e, 10 varieties (dense stripes), L= low diversity, i.e, 2 genotypes (sparse stripes). Letters and color indicate different treatments: H=high diversity (dense stripes), L=low diversity (sparse stripes), R=resistant varieties (red), S=susceptible varieties (blue), and M=50:50 mixture of resistant and susceptible varieties (red and blue). **(B)** Example of random arrangement of the varieties used in one of the plots [with low diversity resistant plots (LR)]. **(C)** Examples of herbivore damage observed in the plots. The majority of damage was caused by sawfly (Symphyta) larvae (top left). Photos by Sanne de Jong.

In 2017, we established a field experiment covering a total area of 130×40m (i.e., similar to the general size of strawberry plantation) at the Alnarp campus of the Swedish University of Agricultural Sciences in southern Sweden (Figure 1A). We divided the area into 60 equally sized plots (size 4m×5m) and applied a full factorial design with two levels of genetic diversity (high and low, with 10 and two varieties per plot, respectively) and three levels of plot resistance (resistant varieties only, susceptible varieties only, and a 50:50 mix of resistant and susceptible varieties in the plots). This resulted in the following treatment combinations: high diversity resistant (HR), high diversity susceptible (HS), high diversity mixture (HM), low diversity resistant (LR), low diversity susceptible (LS), and low diversity mixed (LM) plots (Figure 1A), each of which was replicated 10 times. The position of the varieties within plots was randomized. High diversity resistant and susceptible plots consisted of the maximum 10 varieties of the same resistance class (i.e., all-resistant or all-susceptible) were used in this study, whereas five out of the 10 resistant and five out of the 10 susceptible varieties were randomly chosen for high diversity resistance mixtures. For the low diversity treatment, two out of the maximum 10 varieties per resistance class were randomly chosen for the susceptible and resistant mixture plots, whereas one variety per resistance class was randomly chosen for the low diversity resistance mixture plots (Figure 1B). Each plot contained 40 plants, and two replicates of each variety were grown in the same soil bag (80×30cm; 80% peat+20% pumice; pH=6; 1kg NPK 11.5.18 perm^3^; Emmaljunga Torvmull, Vittsjö, Sweden) resulting in 2400 plants in total.

### Scoring of Damage

To quantify damage caused by chewing herbivores, we counted the total number of leaves with any signs of damage inflicted by herbivores (such as pinholes or damaged leaf edges, Figure 1C) in a plant four times throughout the summer (between June 8th and September 17, i.e., early summer, mid-summer, late summer, and autumn) of 2018. However, the first scoring (early summer) was not used in the statistical analyses due to very low herbivory (0.2±0.69 damaged leaves per plant), but is included in the online repository data set (data uploaded to repository after acceptance). The plant height and width were measured during the last scoring occasion to estimate plant size, that is, photosynthetic area (height×width, cm^2^). As plants in commercial strawberry fields are usually replaced every second year, we revisited the plots and recorded the damage once again in mid-July 2019, before the field was cleared, to obtain preliminary indications of the generality of observed patterns. Because woodland strawberry plants grow new leaves every year, the damage detected in 2019 represents herbivore damage during that growing season and has not accumulated damage from the previous growing season. Therefore, the data set used in the analyses consisted of three scoring occasions from 2018 (from mid-summer to autumn) and one scoring occasion from 2019 (mid-summer).

### Herbivore Community

In order to identify the most common chewing herbivores in the field, we sampled the herbivore community from 20 plots by selecting two replicates of each variety. We covered the whole leaf surface area with a motorized insect aspirator and held this over the leaves for 10sec. In total, we sampled 20 plants with high (more than 10 damaged leaves) and low (maximum two damaged leaves) herbivore damage. Chewing herbivores and leaf damage patterns were identified to the lowest taxonomic level possible based on insect identification guides.

The majority of the chewing herbivores detected in the field were sawfly larvae (Symphyta, *N*=91, i.e., 57%), beetles (Coleoptera, *N*=46, i.e., 29%), and lepidopteran caterpillars (*N*=23, i.e., 14%). Most of the damage was caused by sawfly larvae, *Cladius pectinicornis* (Geoffroy) and *C. difformis* (Panzer). These species of sawfly are known pests of strawberry and occur throughout the whole growing season until October (Alford, 2017).

### Yield

In order to investigate the effect of plot’s resistance and diversity on yield, we harvested all ripe fruits on a weekly basis during May and June during 2019. The fruits were weighed on the day of harvest. For practical reasons, the fruits from four plants of the same variety were pooled and weighed resulting in 10 samples per plot. Yield data were also collected during 2018. However, due to the natural spatiotemporal variation in herbivory, the arrival of herbivores in 2018 occurred after or during the fruit harvest. Therefore, the yield data from 2018 were not analyzed in relation to herbivory.

For statistical models, we calculated the average yield per plant by dividing the weight of the sample box by four (as samples were harvested by combining fruits from four plants of the same variety) to make the data set comparable to leaf damage analyses. Similarly, when using plant size as a covariate in yield analyses, we first calculated the average plant size for each variety in a plot and divided it by four to make the plant size data comparable to yield data.

### Statistical Analyses

#### Herbivore Damage

We analyzed the effects of genotypic diversity and intrinsic resistance on the number of herbivore damaged leaves of each plant in 2018 and in 2019 using a generalized linear mixed model (GLMM, proc. GLIMMIX), with a negative binomial error distribution and log link function. Due to a lack of convergence of a repeated measure model, separate models were constructed for each scoring occasion in 2018. Diversity (*high* or *low*), plot’s resistance (*resistant*, *susceptible*, and *50:50 resistance mixture*), their pairwise interaction, and plant size as a covariate (height×width, cm^2^) were set as independent variables. To control potential edge effects resulting from the shape of the experimental field simulating commercial strawberry cultivation (Figure 1), the positions of the plots in the field were divided into five edge categories (top and bottom, right, left, and middle rows) and used as a random factor in the model. Edge and plot nested with edge were set as random effects to control for spatial nonindependencies of the plot and, thus, plant locations.

#### Associational Effects on Herbivore Damage

To investigate potential associational effects of plant resistance, that is, whether the presence of resistant varieties would protect the susceptible neighbors from damage, we compared whether resistant and susceptible varieties have higher herbivore damage when they are growing among other varieties with a similar resistance class, compared to when they are growing in resistance mixtures. For this, we filtered the data set into two sets based on resistance: to compare the damage on resistant varieties when they grow alone (homogenously resistant) or together with susceptible varieties (resistance mixture) and to compare the damage on susceptible varieties when they grow either alone (homogenously susceptible) or together with resistant plants (resistance mixture). We constructed separate GLMM models with negative binomial error distribution for resistant (growing either among other resistant plants or in a resistance mixture) and susceptible plants (growing either among susceptible plants or in a resistance mixture) during the two growing seasons. We chose the last scoring occasion of 2018 to represent the “damage outcome” of the growing season. The number of damaged leaves was set as a dependent variable, with variety, diversity (high or low), resistance mixture (resistant plants among other resistant plants or in a resistance mixture or susceptible plants among other susceptible plants and in a resistance mixture), plant size (covariate), and the pairwise interactions between diversity and resistance mixture as independent variables. Edge and plot nested within edge were set as random factors.

#### Yield

For analyzing the yield data (fruit weight) from 2019, we used GLMM with a lognormal error distribution. We set diversity, plot’s resistance (resistant, susceptible or their 50:50 mixture), and their interactions as independent variables. The average plant size was used as a covariate. Edge and plot nested within edge were set as random effects.

#### Associational Effects on Yield

As with the analysis for associational resistance to leaf damage, we tested whether the presence of resistant varieties would increase fruit production of susceptible neighbors. Again, we filtered the data to two data sets to compare whether resistant and susceptible varieties have higher yields when growing among other varieties with similar resistance (homogenously resistant or susceptible), compared to when they are growing in resistance mixtures. We constructed separate GLMMs with a lognormal error distribution for yield of resistant and susceptible varieties. Variety, genetic diversity (high or low), resistance mixture (only resistant varieties or in a mixture, and only susceptible varieties or in a mixture), average plant size, and the interaction between plant resistance and diversity were set as independent variables and edge and plot nested within edge as random effects.

In all models, pairwise and three-way interactions of the covariate with plant resistance and diversity were also tested, but as they did not yield any significant result, they were left out of the final model (starting from the three-way interaction). All models were constructed using SAS 7.1 software. The latest Kenward and Roger method (Kenward and Roger, 2009) was used to calculate degrees of freedom.

## Results

The results from all models are reported here but the tables and figures of second and third occasions in 2018 are presented in the supplement (Supplementary Tables S1 and S2, Supplementary Figure S1) due to low herbivore damage and similarity of the results to the other scoring occasions (third from 2018 and one scoring from 2019).

**Figure 2:**
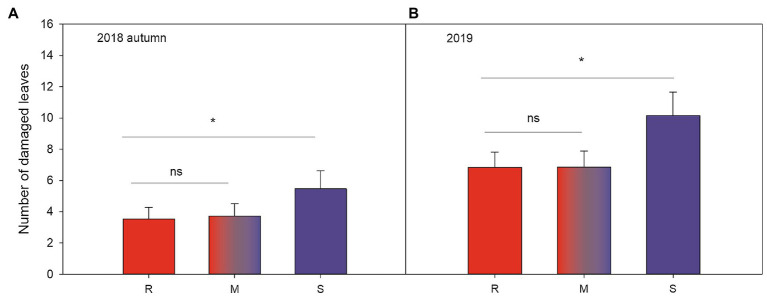
Significant effects (Table 2) of plot resistance (*resistant*, *susceptible* and *50:50 mixture*) during the last scoring occasion **(A)** autumn (mid-September) in 2018 and the only scoring occasion **(B)** during mid-summer (mid-July) in 2019 on number of woodland strawberry (*Fragaria vesca*) leaves damaged by herbivores (mean±SE). Letters along the x-axis indicate different treatments in **(A)**: R=resistant varieties (red), S=susceptible varieties (blue), and M=50:50 mixture of resistant and susceptible varieties (red and blue). Stars indicate statistically significant differences by Tukey’s test (*p*<0.05) described in the text.

**Figure 3:**
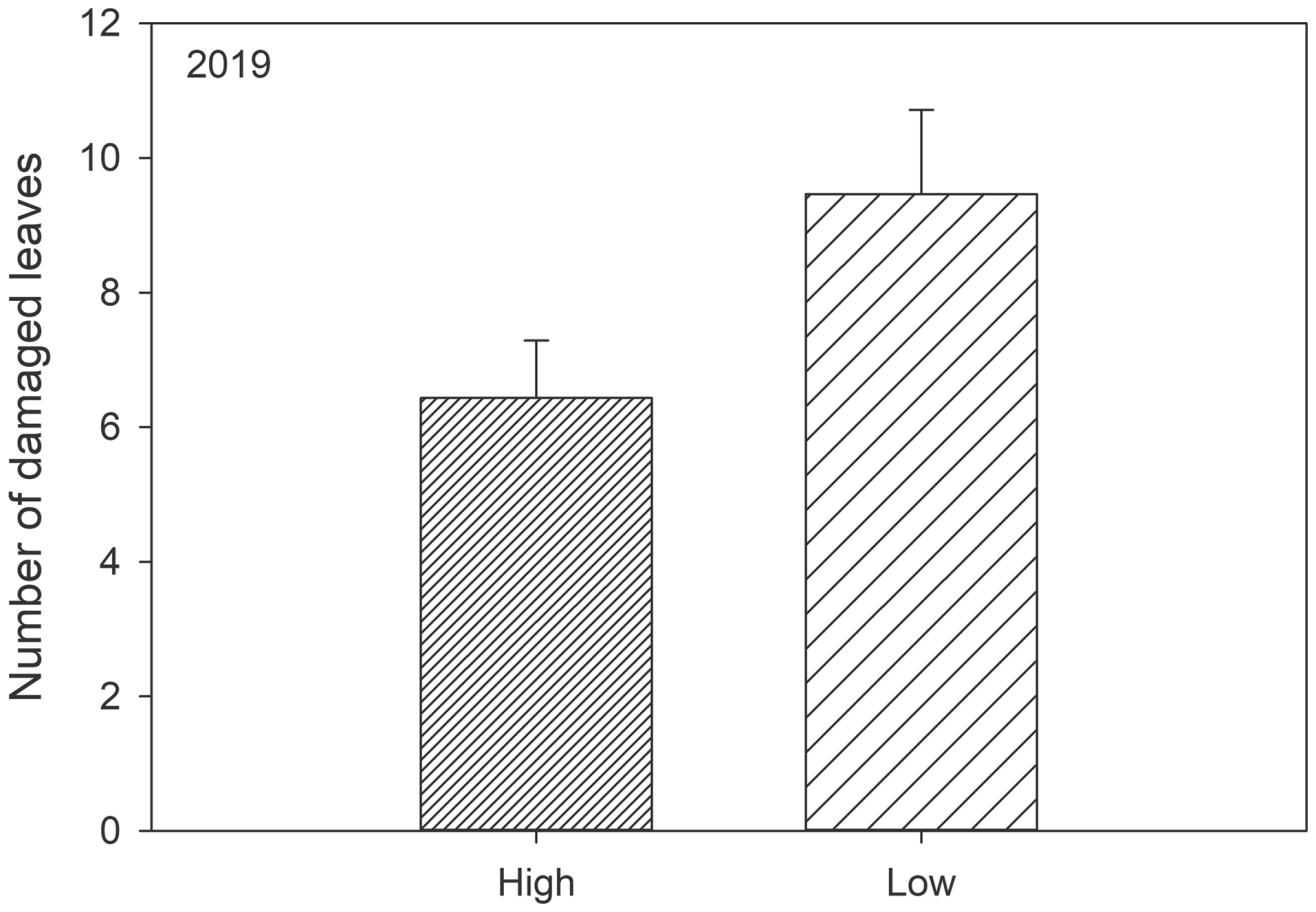
Significant effects (Table 2) of genetic diversity (high, i.e., 10 varieties and low, i.e., 2 varieties) on the number of herbivore damaged leaves of susceptible woodland strawberry (*Fragaria vesca*) plants in 2019.

Plant resistance significantly reduced leaf damage throughout the experiment (Table 2; Figures 2A,B; Supplementary Tables S1 and S2, Supplementary Figures S1A,B): that is, plants growing in susceptible-only plots had more herbivore damage compared to plants growing in resistant (for 2018 last scoring occasion, Tukey-Kramer *F*_1, 49.85_=8.08, *p*=0.007, and 2019 *F*_1, 47.11_=6.89, *p*=0.012) or resistance mixture plots (for 2018 last scoring occasion, Tukey-Kramer *F*_1, 49.36_=6.33, *p*=0.015, for 2019 *F*_1, 46.79_=6.74, *p*=0.013). In contrast, plants growing in homogenously resistant and resistance mixture plots had equally low herbivore damage (for 2018 last scoring occasion, Tukey-Kramer *F*_1, 49.84_=0.12, *p*=0.735; for 2019 *F*_1, 46.47_=0.00, *p*=0.974; Table 2; Figure 2). In addition, plants growing in low genetic diversity plots had significantly more damaged leaves compared to plants growing in high diversity plots, but only during the second growing season (in 2019) when herbivore damage was the highest (Table 2; Figure 3).

**Table 2:** Results of generalized linear mixed models testing the effect of genetic diversity (*high* and *low*, with 10 and two varieties, respectively), plot resistance (consisting only of *resistant*, *susceptible* varieties, or a *50:50 mixture* of the two), and plant size (height×width, cm^2^) on number of herbivore damaged woodland strawberry (*Fragaria vesca*) leaves in 2018 and in 2019.

	2018 last scoring (autumn)				2019 (mid-summer)			
	Num Df	Den Df	F	P	Num Df	Den Df	F	P
Diversity	1	49.43	1.77	0.190	1	45.87	10.0	0.003
Plot resistance	2	49.68	4.82	0.012	2	46.79	4.52	0.016
Plant size	1	2275	101.08	<0.001	1	2164	309.99	<0.001
Diversity×Plot resistance	2	50.1	0.25	0.778	2	48.58	0.80	0.453

Separate models were constructed for each scoring occasion. Plant size (height×width, cm^2^) was measured during the last scoring occasion in 2018. Num Df indicates numerator degrees of freedom, and Den Df indicated denominator degrees of freedom.

In general, plant size had a significant influence on herbivore damage (Tables 2 and 3) as larger plants had more damaged leaves (estimates on a log scale; first scoring occasion 0.0009, 95% CIs 0.0002 to 0.0016, second scoring occasion 0.0022, 95% CIs 0.0018 to 0.0025, last scoring occasion 0.002, 95% CIs 0.0017 to 0.0025, in 2018, and in 2019; 0.0022, 95% CIs 0.0019 to 0.0024).

**Table 3:** Results of generalized linear mixed models testing the effects of associational plant resistance, that is, growing in a resistance mixture (resistant or susceptible variety growing either among varieties of the same resistance class or in resistance mixture plots), genetic diversity of the plot (*high* and *low*, with 10 and two varieties, respectively), and variety (genotype) on the number of herbivore damaged leaves of resistant and susceptible woodland strawberry (*Fragaria vesca*) plants during (A) the last scoring occasion in 2018 and (B) during 2019.

A								
Year 2018								
	Resistant plants				Susceptible plants			
	Num Df	Den Df	F	P	Num Df	Den Df	F	P
Variety	9	922.4	2.78	0.003	9	1002	5.69	<0.001
Diversity	1	31.82	2.20	0.148	1	30.99	0.06	0.807
Plant size	1	1111	27.28	<0.001	1	1085	22.53	<0.001
Resistance mixture	1	31.58	0.0	0.976	1	31.15	6.90	0.013
Diversity×Resistance mixture	1	33.28	0.41	0.526	1	32.9	1.41	0.244
B								
Year 2019								
Variety	9	1120	4.35	<0.001	9	1066	8.73	<0.001
Diversity	1	30.41	7.62	0.010	1	30.61	3.49	0.071
Resistance mixture	1	29.98	0.05	0.818	1	30.83	5.92	0.021
Plant size	1	1100	108.50	<0.001	1	1033	100.57	<0.001
Diversity×Resistance mixture	1	32.06	0.43	0.515	1	32.33	0.05	0.828

Plant size (height×width, cm^2^) was measured at the end of the growing season in 2018. Num Df indicates numerator degrees of freedom, and Den Df indicated denominator degrees of freedom.

### Associational Effects on Herbivory

**Figure 4:**
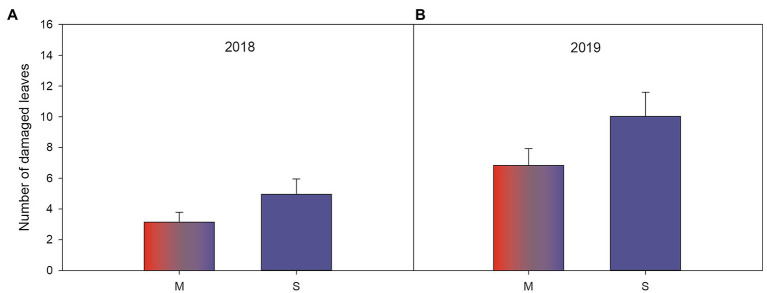
Significant associational effects (Tables 3A,B) of plant resistance (varieties growing either among plants with homogenous resistance level, i.e., in susceptible-only plot, vs. growing in a resistance mixture with both resistant and susceptible varieties) on number of herbivore damaged leaves on susceptible woodland strawberry (*Fragaria vesca*) plants in **(A)** 2018 during the last scoring occasion and **(B)** in 2019. Letters along the x-axis indicate S=susceptible varieties (blue) and M=50:50 mixture of both resistant and susceptible varieties (red and blue).

In both years, susceptible varieties had fewer herbivore damaged leaves when growing in resistance mixtures with resistant plants compared to when they were growing among other susceptible varieties (Tables 3A,B; Figures 4A,B) but there was no such effect for resistant plants (Tables 3A,B). Diversity had no effect on number of damaged leaves during the first growing season (Table 3A), but in the following year (Table 3B), resistant plants in high diversity plots (either when growing among other resistant plants or in the resistance mixture) had significantly fewer damaged leaves (mean 5.33±SE 0.856) compared to resistant plants growing in low diversity plots (mean 8.71±SE 1.31). Overall, variety had a strong effect on herbivore damage even when varieties were compared within the same resistance class (Tables 3A,B).

Larger plants of susceptible (estimate on a log scale 0.0016, 95% CIs 0.0009 to 0.0022) and resistant varieties (estimate on a log scale 0.0017, 95% CIs 0.0010 to 0.0023) during 2018 and 2019 (estimate on a log scale, for resistant: 0.0021, 95% CIs 0.0017 to 0.0025, susceptible: 0.002, 95% CIs 0.0015 to 0.0023) had significantly more damaged leaves (Tables 3A,B).

### Yield and Associational Effects on Yield

Diversity or resistance of the plots did not affect yield significantly (Table 4). Larger plants produced significantly higher yield (Table 4, estimate on a log scale 0.0102, 95% CIs 0.0082 to 0.0122).

**Table 4:** Results of a generalized linear mixed model testing the effects of plot diversity (*high* and *low*, with 10 and two varieties, respectively), plot resistance (*resistant*, *susceptible* and *50:50 mixture of the two*), and average plant size (height×width, cm^2^) measured at the end of the growing season in 2018 on yield (fruit weight, g) of woodland strawberry (*Fragaria vesca*) in 2019.

Yield 2019	Num Df	Den Df	F	P
Diversity	1	49.58	0.34	0.562
Plot resistance	2	49.09	2.06	0.138
Plant size	1	591.7	102.04	<0.001
Diversity×Plot resistance	2	50.15	0.46	0.637

Num Df indicates numerator degrees of freedom, and Den Df indicates denominator degrees of freedom.

Similarly, yield did not differ among resistant and susceptible varieties when they grew in plots among plants with similar resistance level (homogenously resistant or susceptible) or in the resistance mixtures (Supplementary Table S3). Larger plants produced significantly higher yields both among resistant (estimate on a log scale 0.0027, 95% CIs 0.0018 to 0.0035) and susceptible varieties (estimate on a log scale 0.0031, 95% CIs 0.0021 to 0.0040).

## Discussion

We found that increasing the functional diversity of our experimental plots by either growing resistant varieties only or by mixing resistant varieties with susceptible ones reduced herbivore damage more strongly than by increasing genotypic diversity. These results thus show that improving intrinsic resistance of plantations can reduce damage caused by herbivores, and mixing at least some intrinsically resistant plants together with susceptible varieties can help to reduce herbivore damage on susceptible plants. Cultivar mixing by adding intrinsically resistant varieties (here termed “resistance mixtures”) may thus enable the protection of susceptible yet highly productive crop varieties *via* associational resistance. Herbivore damage on resistant varieties remained unaffected regardless of the presence of susceptible varieties, indicating a lack of spillover herbivory from susceptible plants to the less-preferred resistant neighbors. Furthermore, herbivore damage on susceptible cultivars was lower in the presence of resistant neighbors regardless of the number of varieties used. Thus, our results indicate that adding one resistant variety to a susceptible monoculture may be enough to reduce herbivory, thereby minimizing the investment needed for the farmer to switch from monoculture toward more sustainable management.

The amount of natural herbivore damage in the experimental plots was not high enough to reach economically injurious levels (i.e., the damage did not reduce yield). Nevertheless, the results are important from an agricultural perspective, as the results show consistent patterns of reduced pest damage in “resistance mixtures” over two growing seasons, regardless of the level of herbivory. This is a strong indication that “resistance mixtures” confer ecological processes that consistently reduce pest damage in agricultural settings. To our knowledge, this is the first study to simultaneously manipulate different levels of genetic (number of varieties *per se*) and functional (plant and plot resistance) diversity to investigate herbivore damage in an agricultural crop. The importance of these results is not only of relevance for strawberry production, but can be widely applied to different crop species.

Improving the resistance of the plots had a strong and consistent effect in reducing damage whereas increasing the genetic diversity *per se* by planting multiple varieties reduced herbivore damage only during periods of high leaf damage. As intrinsic resistance directly influences herbivore performance and attraction (Weber et al., 2020a), its impact on damage reduction may be more pronounced, whereas the effect of diversity may become evident when certain herbivore pressure or damage threshold is reached. For example, Fernandez-Conradi et al. (2017) found that increased intraspecific diversity reduced herbivore damage on oak only in medium herbivore density but was absent in low herbivore density. Despite that the effect of diversity was weaker, our results indicate that increasing in-field genetic diversity by increasing the number of varieties, as well as improving resistance of the cultivated material by using at least some resistant varieties, can reduce the vulnerability of strawberry plantations to herbivory and, thus, increase cropping security. These results are in line with recent mathematical modeling by Vyska et al. (2016) who showed that cropping security may be further enhanced in resistance mixtures, because the presence of even slightly resistant varieties reduces the likelihood of pest outbreak.

Although the exact mechanisms of the effects of diversity and resistance behind our findings require further studies, both associational resistance and increased habitat complexity (increasing the structural complexity by increasing the number of varieties or increasing the functional complexity by increasing variation in plant traits) are likely to reduce herbivory *via* mutually nonexclusive ways by altering the likelihood of the herbivore encountering its preferred host or likelihood of leaving the food patch (Root, 1973; Duffy, 2002; Civitello et al., 2015; Garrett et al., 2017; Field et al., 2020). These attributes may decrease damage by herbivores through reductions in their colonization rates, reproductive performance, and/or lower consumption efficiency resulting from increases in search effort, lower host encounter rate, and/or diet mixing (Root, 1973; Peacock and Herrick, 2000; Duffy, 2002; McArt and Thaler, 2013; Civitello et al., 2015; Field et al., 2020), thereby relaxing the selection pressure for higher herbivore tolerance. Although theoretically the use of multiple resistant varieties may temporarily result in lower herbivory (as the herbivore would always encounter less-preferred hosts), combining increased genetic diversity (cultivar mixing) with the use of both resistant and susceptible varieties (resistance mixtures) should result in a more comprehensive increase in structural and functional complexity of the plantation and therefore provide better long-term pest control by reducing the likelihood of pests overcoming plant defenses. Although we did not find a synergistic effect of genetic diversity and intrinsic resistance on leaf damage, incorporating both of these strategies should result in higher cropping security because high trait variation confers protection against wider range of pests (e.g., Zhu et a. 2000; Yang et al., 2019).

Besides reducing pest damage, cultivar mixing may also enhance food production if the herbivores are not capable of reaching economically injurious levels (Zhu et al., 2000; Tooker and Frank, 2012; McArt and Thaler, 2013; Reiss and Drinkwater, 2018; Yang et al., 2019). For perennial crops, such as strawberry, management of herbivores is especially important, since the negative effects of pest damage on yield may be manifested over multiple growing seasons (Muola and Stenberg, 2018). In our study, neither resistance nor diversity had significant effects on yield, despite that intrinsic plant resistance is often negatively associated with yield (e.g., Brown, 2002), whereas higher diversity often has the opposite effect (Zhu et al., 2000; Isbell et al., 2017). One possibility is that the natural herbivory was not high enough to reduce the yield, as woodland strawberry appears to be tolerant against leaf damage (Muola and Stenberg, 2018). Damage intensity and timing also depend on temporal fluctuation of the herbivore populations (as observed in the first growing season when herbivores arrived after fruit ripening), and studies over multiple growing seasons are needed to confirm the relative importance of variety and resistance mixing on strawberry yield under different herbivore levels.

To conclude, our results indicate that improving intrinsic resistance of the cultivated material by using at least some resistant varieties can reduce herbivore damage. Cultivar mixing by using intrinsically resistant varieties may thus enable the protection of susceptible varieties, which are typically more high yielding as compared to resistant varieties. Increasing intraspecific genetic diversity of plantations should further reduce pest damage, although such effects may be more seasonally variable, depending on the level of pest damage. The herbivore damage in our study was generally low, and the use of wild (and potentially more genetically diverse or resistant) relative of cultivated varieties may not fully reflect the situation of domesticated plants experiencing economically injurious levels of herbivore damage. Nevertheless, our results provide important insights showing that “resistance mixtures” can offer intriguing opportunities for future plant protection strategies, as they simultaneously increase both the complexity and resistance of the plantations to herbivores. Such a combined approach would probably reduce the need for chemical pesticides in the different crop species, not only strawberries, thus contributing to sustainable food production.

## Data Availability Statement

The raw data supporting the conclusions of this article will be made available by the authors, without undue reservation.

## Author Contributions

All authors conceived the research. JS designed the experiment. JS and SJ collected the data. T-MK analyzed the data. T-MK led the writing of the manuscript with the help of JS and AM. All authors contributed critically to the drafts and gave final approval for publication.

## Funding

This work was made possible by technical assistance from a number of staff affiliated with the SLU Cultivation Unit, the Swedish Infrastructure for Ecosystem Science (SITES Lönnstorp), and the following seasonal assistants: Abdullah Abdulmonem, Helena Edén, Karl-Johan Fabó, Ylva Granberg, Fanny Gustavsson, Alexander Hylander, Astrid Laursen, Johanna Stenberg, Therese Sundin, and Jacob Wlodarski. This work was funded by grants from the Swedish Research Council Formas (project no. 2016-00223) to JS. and the Jenny & Antti Wihuri Foundation (personal grant nos. 00180166 and 777073 to T-MK).

## Conflict of Interest

The authors declare that the research was conducted in the absence of any commercial or financial relationships that could be construed as a potential conflict of interest.

## Publisher’s Note

All claims expressed in this article are solely those of the authors and do not necessarily represent those of their affiliated organizations, or those of the publisher, the editors and the reviewers. Any product that may be evaluated in this article, or claim that may be made by its manufacturer, is not guaranteed or endorsed by the publisher.
