# Supplementary material for: ‘Resistance Mixtures’ Reduce Insect Herbivory in Strawberry (Fragaria vesca) Plantations
Source: Front Plant Sci. 2021 Sep 23;12:722795. doi: 10.3389/fpls.2021.722795 (PMC8494967; doi:10.3389/fpls.2021.722795)

Supplement

‘Resistance mixtures’ Reduce Insect Herbivory in Strawberry (*Fragaria vesca*) Plantations

Tuuli-Marjaana Koski^1,2†^, Sanne de Jong^1^, Anne Muola^1,2^, Daniel B. Amby^3^, Erik Andreasson^1^, Johan A. Stenberg^1^

^1^Department of Plant Protection Biology, Swedish University of Agricultural Sciences, 23053 Alnarp, Sweden.

^2^ Section of Ecology, Department of Biology, University of Turku, 20014 Turku, Finland.

^3^ Department of Plant and Environmental Sciences, University of Copenhagen, 2630 Taastrup, Denmark.

^*^ e-mail: tuuli.marjaana.koski@slu.se

Table S1 Results of generalized linear mixed models testing the effect of genetic diversity (*high* and *low*, with ten and two varieties, respectively), plot resistance (consisting only of *resistant*, *susceptible* varieties or a *50:50 mixture* of the two) and plant size (height × width cm^2^) on number of herbivore damaged woodland strawberry (*Fragaria vesca*) leaves in the first two scoring occasion (middle and late summer) in 2018. Separate models were constructed for each scoring occasion. Plant size (height × width cm^2^) was measured during the last scoring occasion in 2018.

| 2018 | First scoring (mid-summer) | | | | Second scoring (late summer) | | | |
| --- | --- | --- | --- | --- | --- | --- | --- | --- |
|  | Num | Den | F | P | Num | Den | F | P |
| Diversity | 1 | 44.97 | 2.67 | 0.110 | 1 | 49.03 | 3.34 | 0.074 |
| Plot resistance | 2 | 45.27 | 4.67 | 0.014 | 2 | 49.27 | 7.18 | 0.002 |
| Plant size | 1 | 2088 | 7.05 | 0.008 | 1 | 2143 | 183.2 | < 0.001 |
| Diversity × Plot resistance | 2 | 47.17 | 1.38 | 0.261 | 2 | 49.87 | 0.82 | 0.444 |

Num Df indicates numerator degrees of freedom and Den Df indicated denominator degrees of freedom.

Table S2. Results from Tukey’s test for the pairwise comparisons for the significant main effect for the three levels of plant resistance mixtures on leaf damage during the first two scoring occasions in 2018.

| 2018 | First scoring (mid summer) | | | |  | | | Second scoring (autumn) | | | | | | | | | |
| --- | --- | --- | --- | --- | --- | --- | --- | --- | --- | --- | --- | --- | --- | --- | --- | --- | --- |
|  | | | Num Df | Den Df | F | |  | P | | Num Df | | Den Df | | F | | P | |
| Mixed vs resistant-only | | | 1 | 46.77 | 0.08 | |  | 0.779 | | 1 | 49.55 | | 0.04 | | 0.840 | |  |
| Mixed-vs susceptible only | | | 1 | 44.52 | 6.19 | |  | 0.01 | | 1 | 49 | | 10.14 | | 0.003 | |  |
| Resistant vs susceptible | | | 1 | 44.64 | 7.69 | |  | 0.008 | | 1 | 49.26 | | 11.41 | | 0.001 | |  |

Num Df indicates numerator degrees of freedom and Den Df indicated denominator degrees of freedom.

Table S3 Results of a generalized linear mixed model of associational effects, testing the effects of resistance mixture (resistant or susceptible variety growing either among varieties of the same resistance class, i.e., resistant/susceptible only or in resistance mixture plots), genetic diversity of the plot (*high* and *low*, with ten and two varieties, respectively) and variety (genotype) on the yield (fruit weight) of resistant and susceptible woodland strawberry (*Fragaria vesca*) plants in 2019. Plant size (height × width, cm^2^) was measured at the end of the growing season in 2018.

| Yield 2019 | Resistant plants | | | | Susceptible plants | | | |  |
| --- | --- | --- | --- | --- | --- | --- | --- | --- | --- |
|  | Num df | Den df | F | P | Num df | Den df | F | P | |
| Variety | 9 | 276.5 | 14.31 | <0.001 | 9 | 281.1 | 14.99 | <0.001 | |
| Diversity | 1 | 31.0 | 0.55 | 0.463 | 1 | 30.94 | 0.08 | 0.776 | |
| Plant size | 1 | 268.3 | 37.73 | <0.001 | 1 | 249.4 | 41.38 | <0.001 | |
| Resistance mixture | 1 | 30.49 | 0.00 | 0.959 | 1 | 31.4 | 0.36 | 0.554 | |
| Diversity × Resistance mixture | 1 | 31.91 | 0.34 | 0.562 | 1 | 31.33 | 0.80 | 0.379 | |

Num Df indicates numerator degrees of freedom and Den Df indicated denominator degrees of freedom.

Fig. S1. Effects of plot resistance (*resistant*, *susceptible* and *50:50 mixture*) during the first two occasions in 2018 **A**) mid-summer and **B**) late summer on number of woodland strawberry (*Fragaria vesca*) leaves damaged by herbivores (mean ± SE). Letters along the x-axis indicate different treatments in A): R = resistant varieties (red), S = susceptible varieties (blue), and M = 50:50 mixture of resistant and susceptible varieties (red and blue). Stars indicate statistically significant differences by Tukey’s test P < 0.05, (exact values presented in Table S2)


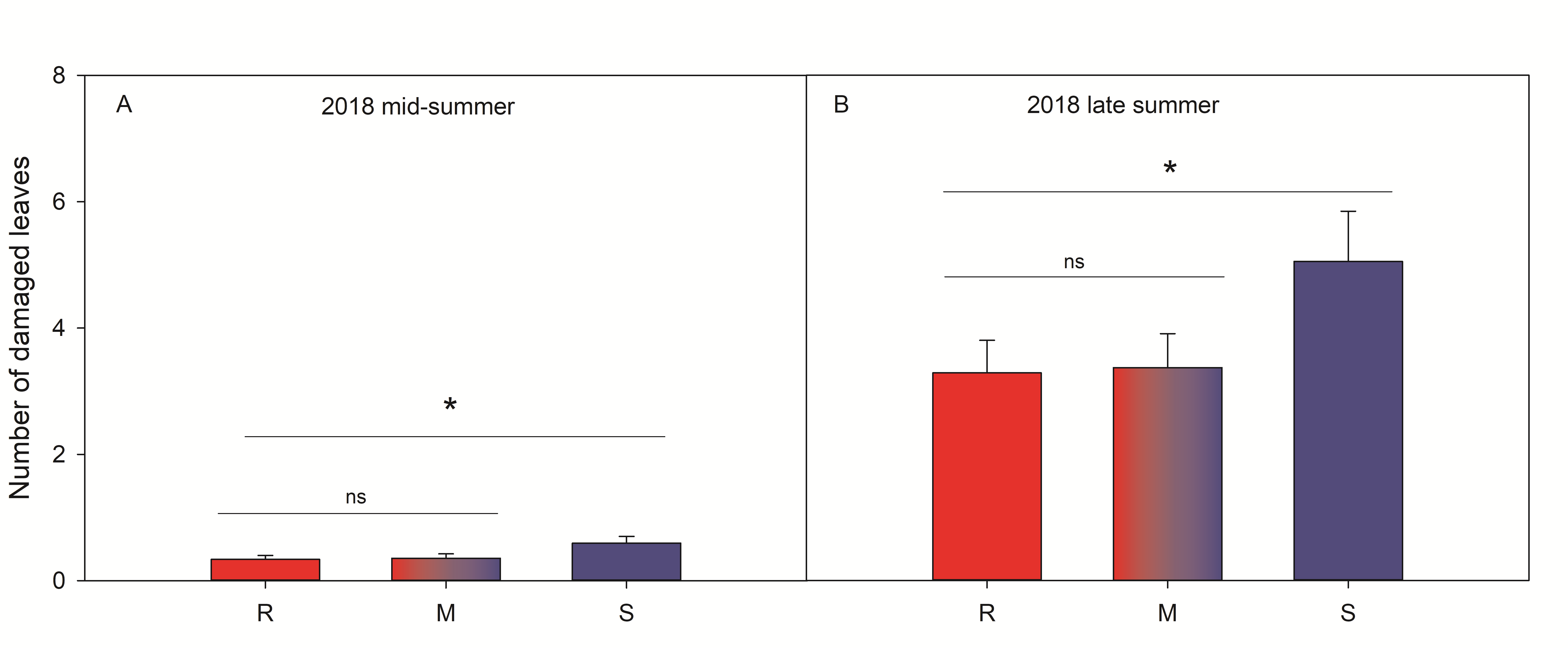

Supplement: Supplementary file 1 [file Data_Sheet_1.docx]
